# Bile reflux index after therapeutic biliary procedures

**DOI:** 10.1186/1471-230X-8-4

**Published:** 2008-02-11

**Authors:** Sedef Kuran, Erkan Parlak, Gulden Aydog, Sabite Kacar, Nurgul Sasmaz, Ali Ozden, Burhan Sahin

**Affiliations:** 1 Gastroenterology Department, Turkiye Yuksek Ihtisas Hospital, Ankara, Turkey; 2 Pathology Department, Turkiye Yuksek Ihtisas Hospital, Ankara, Turkey; 3 Gastroenterology Department, Ankara University Faculty of Medicine, Ankara, Turkey

## Abstract

**Background:**

Therapeutic biliary procedures disrupt the function of the sphincter of Oddi. Patients are potential "bile refluxers". The aim of this study was to assess how these procedures affect the histology-based bile reflux index (BRI), which can be used to reflect duodenogastric reflux (DGR).

**Methods:**

Gastric antrum and corpus biopsies were collected from 131 subjects (56 men, 75 women; mean age, 55.9 ± 15.6 years). Group 1 (Biliary group-BG; n = 66) had undergone endoscopic sphincterotomy, endoscopic stenting, or choledochoduodenostomy for benign pathology; Group 2 (n = 20) had undergone cholecystectomy alone; and Group 3 (n = 6) Billroth II gastroenterostomy. Group 4 (no cholecystectomy; n = 39) had upper endoscopy with normal findings and served as controls. BRI > 14 indicated DGR (BRI [+]). To eliminate confounding effects of *Helicobacter pylori *(*Hp*) infection, comparisons were made according to *Hp *colonization.

**Results:**

Fifty-nine subjects (45%) were *Hp *(+). The frequencies of BRI (+) status in antrum and corpus specimens from *Hp *(-) BG patients were 74.3% and 71.4%, respectively (85.7% for both antrum and corpus for choledochoduodenostomy). Corresponding results were 60% and 60% for Group 2, 100% (only corpus) for Group 3, and 57.1% and 38.1% for controls (BG, Group 2, and Group 3 vs controls – p > 0.05 antrum, p < 0.05 corpus). Fifty-four BG patients had previously undergone cholecystectomy. Excluding those, the rates of BRI (+) in *Hp *(-) BG patients were 75% antrum and 62.5% corpus (p > 0.05 for both vs. Group 2).

**Conclusion:**

Patients who had undergone biliary procedures showed similar bile-related histological changes in both corpus and antrum biopsies, but the changes seen in controls were more prominent in the antrum than corpus. Therapeutic biliary procedures increase the rate of BRI (+) especially in the case of choledochoduodenostomy. Therapeutic biliary procedures without cholecystectomy also increase the rate of BRI (+) similar to that observed in patients with cholecystectomy.

## Background

Duodenogastric reflux (DGR) is a poorly understood gastrointestinal process that is defined as reflux of duodenal contents into the stomach. It is very common for this condition to develop in adults who have undergone gastric surgery, pyloroplasty or cholecystectomy [1,2]. Duodenogastroesophageal reflux (DGER) is the disorder in which material from the duodenum passes into the stomach and the esophagus. Many recent studies have focused on the effects of DGR and DGER. Bile and duodenal contents have chronic noxious effects in both the stomach and the esophagus. Long-term exposure can cause dysplasia, intestinal metaplasia, ulcers and malignancy in the stomach, and Barrett's esophagus and various forms of esophageal malignancy [3].

The underlying mechanisms and motor events involved in DGR are not clear [3,4]. Researchers have looked at the anatomical impacts of pyloroplasty and Billroth II gastroenterostomy, gastric and duodenal motor function, and interactions among these processes. Motor coordination of the stomach, pylorus and duodenum, concentration of duodenal contents, and food intake have been identified as important factors in the development of DGR [4].

It is not clear whether functional and anatomic integrity of the sphincter of Oddi plays a role in DGR. Many patients who need therapeutic biliary procedures undergo endoscopic sphincterotomy, stent insertion, or choledochoduodenostomy and then continue life with a dysfunctional sphincter of Oddi. Such biliary procedures disrupt the control of bile flow into the duodenum, making these patients potential "refluxers". Currently, biliary reflux is usually measured with a fiberoptic spectrophotometer (Bilitec^®^). However, Sobala *et al*. [5] developed a histologic index that identifies DGR based on several findings: edema in the lamina propria, intestinal metaplasia (IM), chronic inflammation, and gastric *Helicobacter pylori *(*Hp*) infection. In this system, a histological bile reflux index (BRI) value above 14 indicates the presence of DGR.

The aim of this study was to investigate how therapeutic biliary procedures, which disrupt the function of the sphincter of Oddi, affect the gastric histology according to BRI.

## Methods

The subjects for this prospective study were the patients who presented to our endoscopic retrograde cholangiopancreatography (ERCP) and endoscopy unit for investigation/treatment of various benign pathologies between April 2003 and October 2003. The reasons for visiting the unit included cholangitis, need for stent exchange, common bile duct stones, and benign biliary stricture. Exclusion criteria were history of alcohol abuse or portal hypertension, current use of a non-steroidal anti-inflammatory drug or any anticoagulant or antiaggregant, and presence of acute pancreatitis. Each subject was assigned to 1 of 4 groups and tissue specimens were collected (details below). Group 1 patients (biliary group [BG]; *n *= 66) had undergone at least one of the following procedures for treatment of benign pathology: endoscopic sphincterotomy (ES), endoscopic stenting, or choledochoduodenostomy. The other patients had undergone cholecystectomy only (Group 2; *n *= 20) or Billroth II gastroenterostomy (Group 3; *n *= 6). Group 4 patients (controls; *n *= 39) had no history of cholecystectomy but had undergone upper endoscopy for dyspepsia or reflux-like symptoms and had normal findings. The BG was divided into 3 subgroups based on the type of biliary procedure performed: ES and stenting (BG1), ES alone (BG2), and choledochoduodenostomy (BG3). All subjects were histologically evaluated for *Hp *infection, and history or not of cholecystectomy was noted for each BG patient. All patients provided written informed consent for the procedures and those who agreed to enter the study were enrolled; the ethical approval for this study was obtained from the Gastroenterology Clinical Council

ECRP was performed using a TJF-240 duodenoscope (Olympus, Japan) and upper gastrointestinal endoscopy was performed with a QX10 esophagogastroduodenoscope (Olympus, Japan). During the procedure, 2 biopsies were obtained from the gastric antrum and 2 from the corpus. A pathologist (GA) who was blinded to the patients' clinical findings assessed each gastric tissue sample according to the above-mentioned BRI system devised by Sobala *et al*. [5]. In this system, an index is derived based on the presence/severity of certain histological parameters: edema (denoted as E in the formula below) in the lamina propria, intestinal metaplasia (IM), chronic inflammation (CI in the formula below), and *Hp *colonization in the stomach. For every specimen, the pathologist assigned a grade from 0 to 3 (representing absent, mild, moderate, or marked, respectively) for each histological parameter. An index value was then calculated using a formula derived from stepwise logistic regression analysis:

*BRI = (7 × E)+(3 × IM)+(4 × CI)-(6 × Hp)*.

According to Sobala and colleagues, a BRI above 14 indicates DGR (defined as bile acid level > 1 mmol/L [the upper limit of physiological reflux]) with 70% sensitivity and 85% specificity.

For analysis, any antrum or corpus specimen with BRI above 14 was identified as BRI (+). For each patient, the average BRIs for the 2 antrum specimens and for the 2 corpus specimens were calculated. The frequencies of antrum BRI (+) and corpus BRI (+) were then calculated for each patient group and for each BG subgroup. To eliminate the confounding effects of *Hp *infection on gastric mucosa, comparisons were made according to *Hp *colonization status. Analysis was also done to test for factors that can affect BRI (+) status, such as patient age and the interval from therapeutic procedure (ES, cholecystectomy, others) to biopsy collection. Group rates of cholecystectomy, IM, and *Hp *colonization were also separately evaluated in relation to age.

Statistical analysis was done using the Statistical Package for Social Sciences (SPSS for Windows, v. 11.0.0). Chi-square was used to compare the group frequencies of BRI (+) status. Regarding the other factors listed above, the Student *t *test was used to compare results for parametric variables and the Mann-Whitney U test to compare results for nonparametric variables. Results were expressed as mean ± standard deviation and *p *values less than 0.05 were considered statistically significant.

## Results

A total of 131 patients (56 men and 75 women; mean age 55.9 ± 15.6 years) were included in the study. Tables 1 lists demographic features and *Hp *colonization status for the patient groups and BG subgroups. Of the 131 patients, 59 (45%) were *Hp *(+). There were no significant differences among Groups 1, 2, 3 and 4 with respect to sex distribution, age or *Hp *(+) status.

**Table 1:** Demographic characteristics and *H. pylori *colonization status in the 4 patient groups and Group 1 subgroups.

	**N**	**Age (y) (mean ± SD)**	**Cholecystectomy**	**No. *Hp*(+) N (%)**
**Group 1**	66	55.4 ± 13.6	54 (81.8)	31 (47)
**BG1 *(ES+stent)***	28	52.7 ± 12.5	23 (82.1)	13 (46.4)
**BG2 *(ES only)***	23	55.2 ± 15.3	16 (69.7)	10 (43.5)
**BG3 *(Choledochoduodenostomy)***	15	60 ± 11.9	15 (100)	8 (53.3)
**Group 2 *Cholecystectomy***	20	64 ± 11.8	20 (100)	10/20 (50)
**Group 3 *Billroth II***	6	62.8 ± 13.6	2 (33.3)	0 (0)
**Group 4 *Controls***	39	51.4 ± 19.3	0 (0)	18 (46.2)

BG: Biliary group. ES: Endoscopic sphincterotomy.

Table 2 shows the frequencies of BRI (+) status (for antrum and corpus specimens separately) in each patient group as a whole and in the proportion of each group that was *Hp *(-). The frequencies of BRI (+) status for the antrum and corpus biopsies from the BG group and the corresponding frequencies for the *Hp *(-) individuals from this group were all higher than the corresponding values in the control group; however, only the differences between the corresponding corpus values were significant. The patients who had undergone Billroth II procedures (Group 3) had the highest BRI (+) frequencies (100% BRI [+] in Group 3 as a whole and same for the *Hp *[-] patients; all 6 in this group were *Hp *[-]). However, analysis excluding this group revealed that the frequencies of BRI (+) status in the corpus specimens from all patients in the BG (48.5%) and in Group 2 (cholecystectomy only; 35%), and from the proportions of these groups that were *Hp *(-) (71.4% and 60%, respectively) were significantly higher than the corresponding rates in the control group (23.1% for whole group, 38.1% for *Hp *[-] portion) (*p *< 0.05 for all). The frequencies of BRI (+) status for the antrum and corpus specimens from the *Hp *(-) individuals in the BG were both higher than the corresponding values in Group 2, but these differences were not statistically significant (71.4% *vs*. 60%, respectively, for corpus; 74.3% *vs*. 60%, respectively, for antrum; *p *> 0.05 for both).

**Table 2:** Frequencies of BRI (+) status for antrum (A) and corpus (C) biopsies in the 4 patient groups, with results listed for all patients in each group and for the proportion that was *Hp *(-).

	**BRI(+) A biopsies in total group (%)**	**BRI(+) A biopsies in *Hp*(-) (%)**	**BRI(+) C biopsies in total group (%)**	**BRI(+) C biopsies in *Hp*(-) (%)**
**Group 1 (Biliary group)**	30/66 (45.5)	26/35 (74.3)	32/66 (48.5)	25/35 (71.4)
**Group 2 Cholecystectomy**	12/20 (60)	6/10 (60)	7/20 (35)	6/10 (60)
**Group 3 Billroth II**	-	-	6/6 (100)	6/6 (100)
**Group 4 Controls**	16/39 (41)	12/21 (57.1)	9/39 (23.1)	8/21 (38.1)
***p***	> 0.05	> 0.05	**0.001**	**0.017**

BRI: Bile reflux index.

Table 3 shows the frequencies of BRI (+) status in each BG subgroup as a whole and in the proportion of each subgroup that was *Hp *(-). The *Hp *(-) portion of the choledochoduodenostomy subgroup (BG3) had the highest frequencies of BRI (+) status (85.7% for corpus, 85.7% for antrum). Most patients in the BG (54 of 66) had undergone cholecystectomy. When these individuals were excluded from the analysis, the frequencies of BRI (+) status in the antrum and corpus biopsies from *Hp *(-) patients in the BG were 75% and 62.5%, respectively. These values were not significantly different from the corresponding results in the *Hp *(-) patients of Group 2 (60% and 60%, respectively) (*p *> 0.05 for both).

**Table 3:** Frequencies of BRI (+) status for antrum (A) and corpus (C) biopsies in the 3 study group (BG) subdivisions, with results listed for all patients in each subdivision and for the proportion that was *Hp *(-).

	**BRI(+) A biopsies in total group (%)**	**BRI(+) A biopsies in *Hp*(-) (%)**	**BRI(+) C biopsies in total group (%)**	**BRI(+) C biopsies in *Hp*(-) (%)**
**BG1 ES+stent**	10/28 (35.7)	10/15 (66.7)	14/28 (50)	12/15 (80)
**BG2 ES only**	11/23 (47.8)	10/13 (76.9)	9/23 (39.1)	7/13 (53.8)
**BG3 Choledochoduodenostomy**	9/15 (60)	6/7 (85.7)	9/15 (60)	6/7 (85.7)
***p***	> 0.05	> 0.05	> 0.05	> 0.05

BRI: Bile reflux index. BG: Biliary group. ES: Endoscopic sphincterotomy.

Differences between frequencies of antrum BRI (+) status and corpus BRI (+) status in each group were also compared. In the BG, these differences were not significant (45.5% *vs*. 48.5%, respectively, for BG as a whole; 74.3% *vs*. 71.4%, respectively, for the *Hp *(-) portion; *p *> 0.05 for both). In Group 2, the frequencies of antrum and corpus BRI (+) status were 60% and 35% for the group as a whole, and 60% and 60% for the *Hp *(-) portion. Neither of these differences was significant (*p *> 0.05 for both). In Group 4, the frequencies of antrum and corpus BRI (+) status were 41% and 23%, respectively, for the entire group and 57.1% and 38.1%, respectively, for the *Hp *(-) portion (*p *> 0.05 for both).

The mean intervals from initial procedure to time of biopsy in BG1 (ES+stenting), BG2 (ES alone), and BG3 (choledochoduodenostomy) were 20.8 ± 21.2 months (range, 3–85 months; median, 13 months), 23.9 ± 35.1 months (range, 0.2–144 months; median, 11 months), and 61.3 ± 77.8 months (range, 2–300 months; median, 41 months), respectively. In Group 2, the interval from cholecystectomy to biopsy was 85.3 ± 112.5 months (range, 1.2–384 months; median, 60 months). Analysis of the BG subgroups and Group 2 revealed no relationship between BRI (+) status in the specimens (antrum and corpus samples analyzed separately) and interval from procedure to biopsy (*p *> 0.05 for both antrum and corpus samples; Mann-Whitney U test).

The mean ages of the 131 subjects were compared according to BRI (+) and BRI (-) status in the antrum and corpus, and were determined as 58.9 ± 15.4 years *vs *52.6 ± 15.5 years for antrum (p = 0.023) and 60.3 ± 14.1 years *vs *52.8 ± 16.1 years for corpus (p = 0.007), respectively. In both categories of results, the BRI (+) status group had a significantly higher mean age.

Other factors linked with BRI (+) status were not found to change with age. When the mean ages of the 131 patients were determined in groups divided according to presence of IM (in antrum and corpus specimens separately [62.6 ± 12.8 years *vs *52.7 ± 16.1 years; 61.6 ± 13.3 years *vs *54.6 ± 15.9 years]), *Hp *colonization (55.2 ± 14.8 years vs 56.5 ± 16.4 years), and history of cholecystectomy (57.3 ± 13.7 years vs 53.9 ± 18.1 years), no significant differences were determined. Analysis revealed that the patients with IM (+) antrum and corpus specimens were older than those with IM (-) antrum and corpus specimens, but the differences were not significant.

## Discussion

Healthy individuals have anatomical and functional barriers that restrict increased intestinal reflux. The pylorus and the physiologically correct angle between the duodenum and the bulbus are the main anatomical factors. Antroduodenal motility, pyloric motility, and coordination of these activities are the main functional factors. The exact mechanisms of DGR are not known. This type of reflux is a physiological phenomenon that occurs postprandially and during sleep [6-9], but in some situations it becomes pathologic. This can occur, for example, after gastric surgery [10,11] or cholecystectomy [12-15]. The healthy gastrointestinal tract has numerous mechanisms that defend against secretions, which are normally found in the lumen. However, proximal reflux of duodenal juice can damage unprotected mucosa [16]. Research has revealed much evidence that duodenal juice has noxious effects when it occurs in abnormal sites or accumulates in massive amounts [17-23]. As noted, therapeutic biliary procedures impair the function of the sphincter of Oddi, leading to uncontrolled flow of bile into the duodenum. These patients are prone to DGR. Only a few studies have evaluated the effects of therapeutic biliary procedures on DGR and the results are conflicting. Fountos *et al*. [24] investigated using scintigraphy and found that DGR is common after biliary surgery (cholecystectomy or cholecystectomy-choledochoduodenostomy) and procedures such as ES. They did not evaluate the *Hp *status of their patients. Tritapepe *et al*. [25] also investigated with scintigraphy and observed that choledochoduodenostomy did not increase DGR, whereas transduodenal sphincteroplasty did. Di Vita and colleagues [26] used scintigraphy to assess DGR in 23 patients (sphincterotomy performed in 16; choledochoduodenostomy in 7). They detected DGR in only 1 patient who had undergone sphincterotomy, but in almost all those who had undergone choledochoduodenostomy. In all these studies, scintigraphy was used to assess DGR and the impact of *Hp *infection was not evaluated. In our research, we used histological BRI to assess DGR. Our data indicate that therapeutic biliary procedures increase the frequency of BRI (+) status especially in choledochoduodenostomy.

It is difficult to diagnose DGR. Reports note conflicting results with respect to histopathologic changes in the gastric mucosa of these patients. Stein and colleagues [27] investigated the diagnostic value of gastric histology relative to degree of DGR and found a poor correlation. In contrast, Dixon *et al*. [17] used the histological BRI and detected a strong correlation between this index and gastric bile acid levels. Dixon and other co-workers (Sobala *et al*. [5]) had introduced their BRI system in a previous study, detailing the derivation of this index by stepwise logistic regression analysis of histological grades of various factors (edema, IM, chronic inflammation, *Hp *colonization). The later work by Dixon *et al*. [17] revealed that patients with Barrett's esophagus exhibit more evidence of bile-related gastritis than those with gastroesophageal reflux disease or non-ulcer dyspepsia. In an even more recent study, Dixon *et al*. [18] found that IM at the cardia was associated with histological evidence of bile reflux into the stomach. The Bilitec^® ^device is the best method for monitoring bilirubin levels in the esophagus; however, interpretation of gastric bilirubin is more complex and Bilitec^® ^is not as accurate in this setting [28-30]. Also, the value of measuring bilirubin is that high bilirubin levels indicate possible histologic changes in affected mucosa. The BRI is derived from observed changes in tissue histology and is, thus, an important tool that can reflect mucosal changes caused by bilirubin.

In our study, the frequencies of BRI (+) status in antrum and corpus biopsies from BG patients were similar to the corresponding rates in Group 2 (cholecystectomy only). Most of the BG patients (54 of 66) had undergone cholecystectomy. The BG subgroups who had undergone ES or stenting in addition to cholecystectomy had somewhat higher frequencies of BRI (+) status than Group 2 (cholecystectomy only), but these differences were not significant. Only the choledochoduodenostomy subgroup (BG3) had significantly higher frequencies of BRI (+) in both corpus and antrum biopsies when compared to Group 2. After choledochoduodenostomy, the opening of the common bile duct is located more proximal to the bulbus than its original site, and the angle at which it opens into the duodenum is changed. This anatomical alteration can lead to increased frequency of BRI (+) status. In Group 1 (BG), 54 patients had undergone cholecystectomy in addition to other therapeutic biliary procedures and the other 12 had undergone the therapeutic procedure(s) alone (no cholecystectomy) (Table 1). Within the latter group of 12, the frequency of BRI (+) status in the individuals who were *Hp *(-) was similar to that observed in the *Hp *(-) portion of Group 2. These results suggest that performing therapeutic biliary procedures in patients who have already undergone cholecystectomy increases the likelihood of BRI (+) status, but therapeutic biliary procedures alone also increased the BRI (+) status as much as observed in patients who had cholecystectomy. Thus, therapeutic biliary procedures are a risk factor for increased frequency of BRI (+) status.

We also looked at relationships between various parameters in this study. The analysis indicated that, for patients who undergo ES, endoscopic stenting, choledochoduodenostomy or cholecystectomy, there is no relationship between the time at which the procedure is performed and BRI (+) status. In contrast, our results for age identified this as an important factor in the histological BRI system. The patients with BRI (+) status were significantly older than those who were BRI (-). A few previous studies have tested links between age and DGR. Bollschweiler *et al*. [31] evaluated bile reflux into the stomach and esophagus in a population of volunteers older than 40 years. They found no significant difference in reflux into the stomach when patients were grouped according to age (younger [median age 25 years] *vs*. older [median age 51 years]). Byrne and co-workers [32] also observed no link between age and DGR. In both of these studies, the Bilitec^® ^method was used to measure levels of DGR. In contrast, our findings using the histological BRI system indicated that BRI (+) status is more common in older than in younger individuals.

Our findings related to age made it essential to evaluate factors that can change with age, such as *Hp *colonization status, history of cholecystectomy, and presence of IM. We observed no significant differences in mean age when our 131 subjects were divided according to *Hp *colonization status (*Hp *[+] *vs. Hp *[-]) or according to history of cholecystectomy. When patients were categorized according to presence of IM in antrum and corpus specimens (separately), there were no significant differences between the mean ages of the groups with and without IM in both the antrum and the corpus.

## Conclusion

In conclusion, DGR is an important gastroenterological process that needs to be more fully understood. The goal of this study was to evaluate how various therapeutic biliary procedures affect the histology of the stomach. Our results indicate that therapeutic biliary procedures, especially choledochoduodenostomy, can be a risk factor for increased DGR. We also observed that, compared to controls, patients who had undergone biliary procedures showed similar bile-related histological changes in the corpus and the antrum. One would normally expect to find a higher level of bile in the antrum than in the corpus. Interestingly, in the groups/subgroups in our study that were at higher risk for increased DGR (*i.e*., patients with history of cholecystectomy, or those who had undergone cholecystectomy plus therapeutic biliary procedures), the frequency of increased DGR in the corpus was similar to that in the antrum. This suggests that bile reflux causes more marked histologic disturbances in the proximal stomach than in more distal areas. Our results also suggest that older patients and those who are *Hp *(-) are at higher risk for developing increased DGR.

## Competing interests

The author(s) declare that they have no competing interests.

## Authors' contributions

All authors read and approved the final manuscript.

S K made substantial contributions to conception and design of the study, and acquisition, analysis and interpretation of data, and was also involved in drafting the manuscript.

E P made substantial contributions to conception and design of the study, acquisition of data, and revision of the manuscript.

G A conducted pathological reporting and contributed to conception and design.

S K made substantial contributions to conception and design, was involved in drafting the manuscript, and provided critical revision of scientific content.

N S was involved in providing critical revision of scientific content.

A O was involved in providing critical revision of scientific content.

B S was involved in providing critical revision of scientific content, and gave final approval of the version to be published.

## Pre-publication history

The pre-publication history for this paper can be accessed here:


